# Co-design of a walking activity intervention for stroke survivors

**DOI:** 10.3389/fresc.2024.1369559

**Published:** 2024-06-04

**Authors:** H. Wittink, C. van Gessel, J. Outermans, T. Blatter, M. Punt, R. van der Lugt

**Affiliations:** ^1^ Research Group Lifestyle and Health, Utrecht University of Applied Sciences, Utrecht, Netherlands; ^2^ Co-design Research Group, Utrecht University of Applied Sciences, Utrecht, Netherlands

**Keywords:** stroke, behavior change, walking performance, co-design, toolkit, sensors

## Abstract

**Introduction:**

Stroke survivors may not maintain gains made in gait performance after task-oriented circuit training. Behavior change interventions may enhance the long-term adoption of physical activity. This study uses a co-design methodology to develop an intervention and tools to facilitate physical and exercise therapists in supporting an active lifestyle in stroke survivors, which is defined as a lifestyle that integrates daily walking performance with day-to-day activity.

**Objectives:**

(1) To describe the insights generated during the co-design process; and (2) To describe the tools that were developed during the co-design process.

**Methods:**

A multidisciplinary team consisting of staff members of the Royal Dutch Society for Physical Therapy, exercise and physical therapists specializing in neurorehabilitation and conducting task-oriented circuit class training in primary care settings or day therapy centers within residential care facilities, stroke survivors and their carers, experts in measuring movement behavior in stroke survivors, a company specializing in manufacturing sensors and related software, behavior change specialists, and co-designers all collaborated in a three-stage (define, develop, and deliver) co-design process.

**Results:**

In the design process, the team iteratively developed a prototype accelerometer system for measuring walking performance with a feedback function for stroke survivors and their therapists and a prototype toolbox for therapists to support the facilitation of behavior change in their stroke survivors.

**Discussion:**

This study shows how co-design can be applied to develop interventions for stroke survivors. Both the prototype system for measuring walking performance and the toolbox incorporate behavior change techniques to support a more physically active lifestyle in stroke survivors. Further research will investigate the feasibility of the intervention.

## 1 Introduction

Stroke is the second-leading cause of death and a major cause of disability worldwide (1). Those surviving a stroke may often experience residual functional disabilities, emotional problems, cognitive deficits, and poststroke fatigue. Physical activity after a stroke is essential for aiding the process of recovery, improving general health, and reducing future stroke risk (2). In addition to the physical health benefits of being active, physical activity may also offer psychological benefits, such as reducing the risk of poststroke depression (3, 4), which is highly prevalent in stroke survivors (5) and is associated with an increased risk of mortality (6).

Walking has been shown to be an especially important form of physical activity for stroke survivors (7). Although a majority of stroke survivors are able to walk independently six months after stroke (8), many cannot walk with sufficient speed, have to use walking aids, and are restricted in their ability to move around both indoors and outdoors(9). Regaining walking ability, therefore, is a common goal in rehabilitation, as it is associated with independence and participation (10, 11).

In the Netherlands, a commonly used strategy to improve gait and gait-related activities is to engage stroke survivors in task-oriented circuit training. Task-oriented circuit training is a high-intensity progressive motor learning–based intervention wherein a small group of stroke survivors exercise functional motor tasks on different workstations (12). Significant effects have been found for gait velocity, gait endurance, balance, and strength of the lower extremities (11, 13–15) but not for usual walking performance in chronic stroke survivors (16). Stroke survivors are often predisposed to a sedentary lifestyle, walk less than virtually any other clinical population (17), and take 50% fewer steps every day than their age-matched peers (18). Not surprisingly, the positive results of these programs gradually disappear after discharge from the program (19–21). It, therefore, seems of paramount importance to develop an intervention that will facilitate the maintenance of the positive (capacity) gains made during task-oriented circuit training by improving walking performance. Walking performance refers to actual walking activity in the usual environment. One approach could be to combine task-oriented circuit training with a behavioral intervention to improve walking performance outside of therapy sessions. Behavioral interventions make use of behavior change techniques (BCTs), which are the smallest components of behavior change interventions that, on their own, in favorable circumstances, can bring about positive behavior change. Behavior change techniques have been described in the Behavior Change Technique Taxonomy (22), which offers a reliable method for specifying, interpreting, and implementing the active ingredients of interventions to change behaviors that can be used by researchers and practitioner communities (23). The taxonomy includes 93 BCTs clustered into 16 groups that include, for instance, goals and planning, feedback and monitoring and social support.

A systematic review of interventions for improving community ambulation found insufficient evidence to establish the impact of community ambulation interventions (24). Current evidence on effective intervention strategies to target stroke survivors’ physical activity behavior in general and community walking performance specifically is still very limited (25, 26), and there is a paucity of information on behavioral interventions to improve community walking performance (21). A systematic review of interventions to promote long-term participation in physical activity after stroke (21) concluded that few studies included self-regulatory techniques, such as goal setting, planning, monitoring, and feedback, despite their proven effectiveness in other clinical populations. Goal setting in stroke rehabilitation has been identified in a review and previous research (7, 27, 28) as being important for recovery and having a positive influence on stroke survivors’ perceptions of self-care ability and engagement in rehabilitation. In line with the previous review, another systematic review (29) found nine promising BCTs: information about health consequences; information about social and environmental consequences; goal-setting behavior; problem-solving; action planning; feedback on behavior; biofeedback; unspecified social support; and a credible source to improve physical activity behavior. There is some evidence to suggest that individual-tailored counseling to address barriers to physical activity and the provision of motivational support for physical activity after a stroke lead to better engagement in physical activity after the end of rehabilitation (21). A scoping review concluded that technologies, combined with behavior change theory, have been applied in a variety of ways with encouraging results (30). A recent Cochrane study (31) concluded that there was not enough evidence to support the use of activity monitors to increase physical activity after stroke. Feedback on walking performance by means of accelerometers, in conjunction with professional advice and support, however, has been shown to have positive effects on home and community walking (32, 33).

Due to the paucity of evidence for effective behavior change interventions in stroke survivors, we chose co-design, also known as participatory design, as a method for our study. When there is a lack of concrete evidence for a specific intervention in clinical care, healthcare providers often rely on tacit knowledge as a starting point. This tacit knowledge can include insights gained from previous patient cases, understanding subtle clinical cues, and recognizing patterns that may not be explicitly outlined in the research literature. Tacit knowledge is not limited to healthcare providers; patients and their carers also possess their own form of tacit knowledge based on their experiences, perceptions, and personal insights related to their health and well-being. Co-design processes create opportunities for participants to share their tacit knowledge, experiences, and insights to arrive at solutions when no evidence exists. Co-design is an approach that involves multiple participants in the process of designing products, services, or interventions. The term “participant” reflects individuals who have a vested interest in the design process and can include healthcare providers, patients, caregivers, administrators, designers, researchers, and any other relevant parties who bring their expertise, perspectives, and experiences to the collaborative design process. The co-design approach enables participants to work together for the improvement or creation of shared solutions that are fit for purpose and are based on the real needs and desires of those who are their direct beneficiaries. This ensures shared ownership of solutions and their delivery and dissemination (27). Consequently, participants can design interventions that seamlessly integrate into existing workflows, leading to improved satisfaction and acceptance (34). Because of the emphasis on the involvement of stakeholders, co-design is often context-specific, and the results may not always be generalizable to other settings or populations. For generalizability, it is often necessary to conduct further research or adaptation to validate and refine the co-design outcomes in different settings.

The goal of this Active Living after Stroke (ACTS) study was to develop a behavior change intervention to improve walking performance in stroke survivors as a form of task-specific aerobic training. The aim of this paper is (1) to describe the insights generated during the co-design process and (2) to describe the tools that were developed during the co-design process.

## 2 Materials and methods

The ACTS study uses a Participatory Action Research (PAR) design approach to co-design the intervention. In this study, we build on the previous literature and our previous studies on walking performance after stroke (35) and accelerometer use for measuring walking performance (36, 37).

### 2.1 Participants

The ACTS project consortium consisted of a multidisciplinary development team consisting of eight practicing female and two male exercise/physical therapists specializing in neurorehabilitation and conducting task-oriented circuit class therapy in primary care settings or day therapy centers within residential care facilities, one male member of the Royal Dutch Society of Physiotherapy, three female and two male co-designers, three male experts in behavior change, two male engineers specializing in manufacturing movement sensors and related software, and one female patient representative*.* The patient perspective was further represented throughout the design process through the use of personas (38). This lead to the inclusion of the patient's perspective, without burdening patients with travel and meetings. Personas were built on previous research, interviews, and observations. In preceding qualitative research (39), stroke survivors indicated a number of factors ranging from the physical and social environments, social influence, attitude, self–efficacy, and capability that could act as either barriers or facilitators to walking outdoors. The often-mentioned barriers were the fear expressed by carers and partners related to falls by patients. Facilitators often had a purpose, such as going for coffee at their neighbors’ place or going to the post office. Semistructured interviews were used to probe what people *say and think* (explicit knowledge**)**, and observations during fit-stroke sessions were used to understand what people *use and do* (behavior and observable knowledge). Co-designers attended two fit-stroke sessions during which they spoke with stroke survivors, their carers, and therapists and observed them in the clinic. From the interviews, observations, and previous research (39), scenarios were built of how stroke survivors, carers, and physical therapists interact to arrive at five different socionas. Socionas consist of a visual description of the dynamics in a system of people [a stroke survivor, a therapist, and a carer (40, 41)] to consider the barriers and facilitators for the three participant groups (stroke survivors, caregivers, and therapists). Socionas were linked to the behavioral lenses (42, 43) (How to inform and change opinions?—How to motivate and enable? How to realize and be aware? How to change habits and impulses? See Supplementary Appendix 1). The behavioral lenses were developed based on relevant behavioral science theories and were designed to understand the behavior of the target group by asking questions such as “Is this an automatic behavior”? When and where do these behaviors occur? What circumstances seem to promote this behavior? This model incorporates the most promising BCTs that were identified in a review of the literature (22).

All consortium partners assisted with the recruitment of participants when this was required for specific co-design activities, including stroke survivors and their carers (e.g., partners and children), physical therapists, designers, researchers, and students.

### 2.2 Ethics

The University of Utrecht Human Research Ethics Committee confirmed that the Medical Research Involving Human Subjects Act (WMO) did not apply to this study, and all participants gave written informed consent. All participants were treated according to the guidelines of good clinical practice (44).

### 2.3 Procedure

Prior to the co-design sessions, interviews were conducted with stroke survivors, their carers, and therapists to gain a deeper understanding of the problem.

Participants were then involved in a series of three co-design stages (define, develop, and deliver) that were held at one site (HU University of Applied Sciences in the heart of the city of Utrecht in the Netherlands) between May 2017 and December 2017. A co-design core team was responsible for the planning and preparation of the co-design activities. This core team consisted of three researchers: one female co-designer, a female academic physical therapist, and a male expert in behavior change who was also part of the consortium. A typical co-design session took 4 h and involved 10–15 participants, mainly physical and exercise therapists. Patients and their carers were represented by the socionas. A team of two co-designers prepared the co-design sessions by formulating desired outcomes and setting up system maps to present the data. During the co-design sessions, these designers operated as workshop facilitators and used various assignments (e.g., prioritizing ideas for prototype concepts) to work toward the desired outcomes in an open atmosphere where everyone was invited to actively participate. All written co-design session data (e.g., homework post-its, drawings, notes) were collected, discussed, and analyzed during core team evaluation meetings directly after the co-design session.

#### 2.3.1 Co-design stage 1: define

In co-design terminology, the co-design stage is called “Empathize.” During this stage, the team focuses on understanding the problem deeply and gaining insights into users’ needs. In this study, the purpose of this stage was to generate insights into the interrelationships between stroke survivors, carers, and therapists related to behavior change toward increasing physical activity. The homework assignment prior to this first co-design session consisted of requesting participants to provide a theoretical insight, a clinical insight, and an insight from a stroke survivor on how to get stroke survivors to be more physically active at home after rehabilitation, in addition to a dream for their future. Unfortunately, many patients had no immediate desire to get more active, as they felt they were too old, too tired, in too much pain, or too limited in their mobility, thereby not grasping the importance of maintaining or improving their health by walking more. Patients indicated that they felt the need to be stimulated to get moving again in order to be able to do more, and that it was fine to increase their walking activity as long as it was for a valuable goal and they were not walking “just to walk.” The strategies mentioned to stimulate walking were: compliments and rewards, walking in a group, structuring the week by making a schedule, showing progress, and being aware of the long-term benefits of walking more.

During this co-design session, two situations, a successful one and a difficult one, were explored to deepen insights using the socionas. Therapists were asked to list the elements that influence stroke survivors’ walking behaviors and how they relate to the behavioral lens(es), including triggers and effects of the behavior, and to then use the behavioral lens(es) to ideate ways to change the current behavior to the target behavior they (and the stroke survivor) are aiming for. In this discussion phase, participants included and explicitly mentioned the aspects that they regarded as most important (know, feel, and dream), which allowed the research team to find any blind spots (45). The discussions were audio-recorded and transcribed, and field notes were taken by the researchers.

#### 2.3.2 Co-design stage 2: develop

This co-design stage is called the “ideation phase.” In the ideation phase, the team generates creative ideas and potential solutions. Effective teamwork encourages risk-taking, encourages wild ideas, and ensures that no idea is dismissed prematurely. In this session of the study, the purpose was to develop different answers to the problem. Participants were e-mailed homework to enrich the five socionas with real-life stroke survivor stories and to envision treating these stroke survivors. Participants were also asked to combine the homework ideas with an accelerometer (BCT feedback on behavior) and build a morphological chart. A morphological chart is a tool for generating ideas in a systematic manner to come up with unexpected alternatives for complex designs (who, what, when, where, and why chart). During the co-design session, the scenario and the idea were played out by the stakeholders. The participants then experimented with various prototypes.

#### 2.3.3 Co-design stage 3: deliver

This co-design stage is also called “prototyping.” During prototyping, the team starts creating tangible representations of their ideas. Effective teamwork ensures that prototypes evolve rapidly, incorporating different perspectives and skills, leading to better solutions. In this study, the purpose of the third co-design session was to explore and try out interventions. An accelerometer with a feedback screen (BCT feedback on behavior) for the stroke survivor was developed, with a software system designed to upload the accelerometer data to the physical therapist's computer. The prototype accelerometer was combined with the behavioral lenses and the scenarios of the five socionas and presented in an intervention principles poster (see Supplementary Appendix 2). Here, behavior change techniques were introduced (see Supplementary Appendix 2). In this co-design session, three interventions, (1) interaction with the stroke survivor, getting to know the stroke survivor; (2) accelerometer and screen; and (3) inspiration to walk at home (getting the social environment involved) were explored, improved, and enriched. The participants were asked to share their thoughts and experiences, and interventions were played out for all roles.

In addition, two participants (physical therapists) discussed the interventions with their patients, and two researchers discussed the interventions with two patients and their carers outside the co-design setting.

### 2.4 Data analysis

All interviews and sessions were recorded and transcribed. Field notes were taken by the researchers. The data analysis was iterative, using the knowledge created to inform action and the next stage of co-design. Collaborative analysis was used to generate data as a basis for reflection on commonalities, patterns, differences, underlying causes, or potentials on an ongoing basis (46). All information from within and without the co-design sessions was synthesized by the core team. A toolbox was created combining the accelerometer with the feedback screen, the behavioral lenses, tools for getting to know the stroke survivor (generative communication tools to explore a stroke survivor's day routine, home, outdoor environment and social network), and tools for inspiring the patient to walk at home, setting (valuable) goals, and evaluation forms (see Supplementary Appendix 3).

## 3 Results

Interviewed stroke survivors, their carers, and their therapists reported that there were large differences between stroke survivors. Stroke therapists, therefore, indicated a need to know the stroke survivors and their context very well and identified a need for objective information on walking performance to be able to provide personalized coaching. Walking behavior was perceived to be important by stroke survivors, but mainly for social activities. Carers were identified as those who needed to be involved, as therapists had no insight into walking performance at home. Participating stroke therapists discussed the range of clinical presentations in stroke survivors. They identified physical impairments, environmental limiting factors, a lack of knowledge of the benefits of being active, (lack of) motivation, and (old) habits as potential barriers. An emergent theme from the therapists was that stroke survivors require individualized intervention approaches for which the interaction between stroke survivors, carer, and therapist is of utmost importance for the outcome of the intervention; consequently, communication skills are important. Another emergent theme was the unfamiliarity of the therapists with the use of behavior change techniques and how to choose which approach to use with which stroke survivor. It has been suggested that improving walking performance is not a goal in itself but a means to achieve valued goals. Providing visual feedback on the target behavior (i.e., steps walked per day) and the valued goal could motivate stroke survivors and their carers. Motivation to improve walking performance was thought to come from progress and compliments. Therapists would, therefore, need to be able to monitor stroke survivors’ progress remotely.

### 3.1 Co-design stage 1: define

Various scenarios for stroke survivors were sketched:

(a) Some stroke survivors have never been physically active or have habits that are quite sedentary (opportunity and attitude). Some stroke survivors know nothing about physical activity and do not know if this is good for them. Or they may have negative thoughts about physical activity, think it is bad for them, and are resistant to being more active (attitude and social influence).
(b) Some stroke survivors do not want to be active or cannot be. They feel insecure or frustrated and lack the motivation or skills to become more active (efficacy and ability).
(c) Some stroke survivors find it too difficult to become more active, or they have tried it and found it too difficult to stay active and to keep remembering to keep active (cognitive and physical ability).
(d) Some stroke survivors do not realize how inactive they really are; they think they are sufficiently active or make up excuses (cognitive ability).

**Figure 1:**
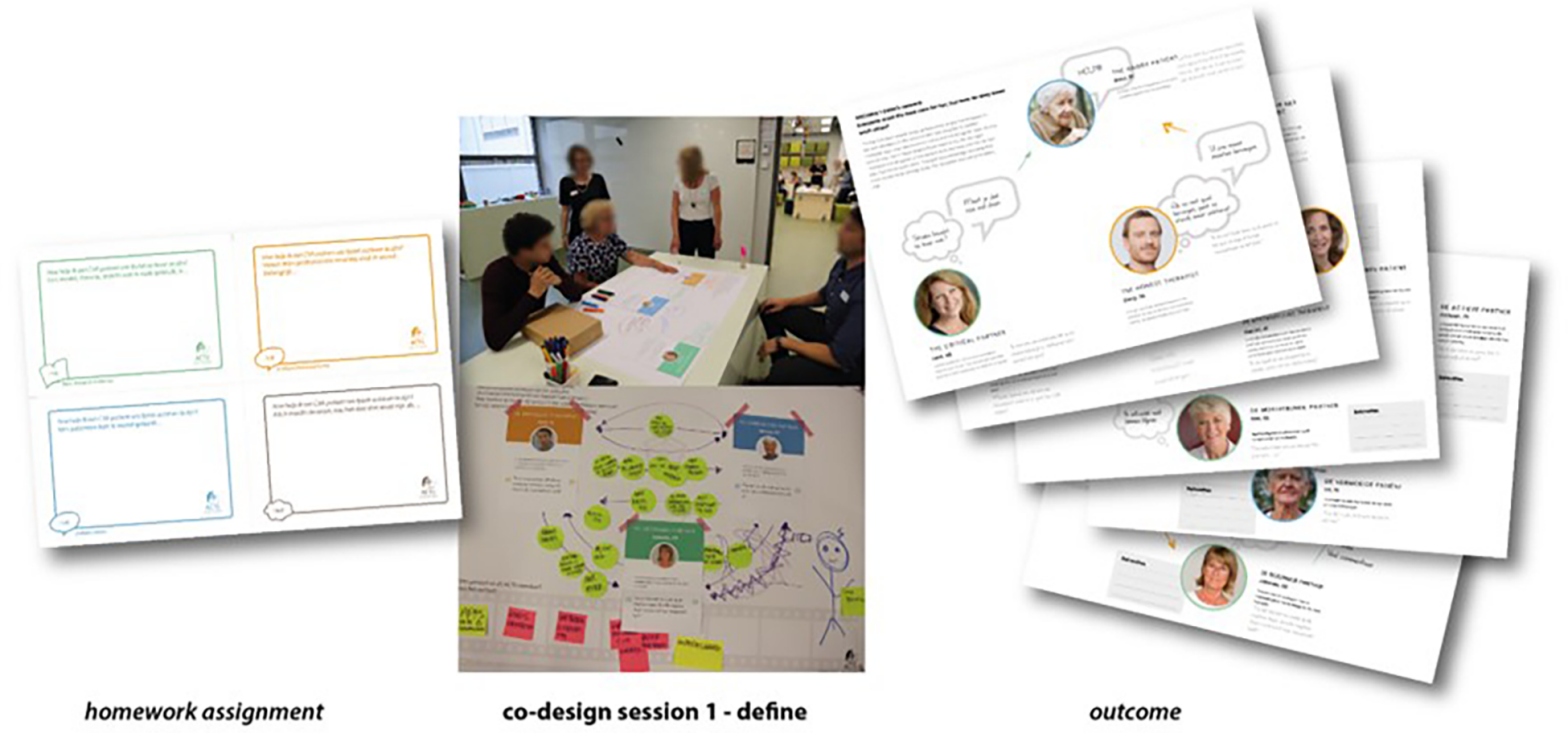
Co-design session 1—define.

All these yielded questions such as how more structure at home can be offered, how stroke survivors can be inspired to move at home, and how carers can be better supported. Some insights emerged. To increase the intrinsic motivation of the stroke survivor, activities must be of value (as in support-valued goals); they must be both feasible and fun. In addition, motivation also comes from progress, compliments, and social support. Insights were that a personal behavior change plan connects the therapist, stroke survivor, and carer, based on objective measurements of the accelerometer on walking activity. For a personal behavior change plan, goals are set by the stroke survivor and therapist together and subsequently broken down into small steps. Please see Figure 1.

### 3.2 Co-design stage 2: develop

Insights were that the feedback from the monitor of the accelerometer needs to link to the valuable goals for the stroke survivors and that the daily measurements need to be fed back to the therapist. The therapist can then give feedback on progress toward the set goals and make suggestions on how to achieve them.

**Figure 2:**
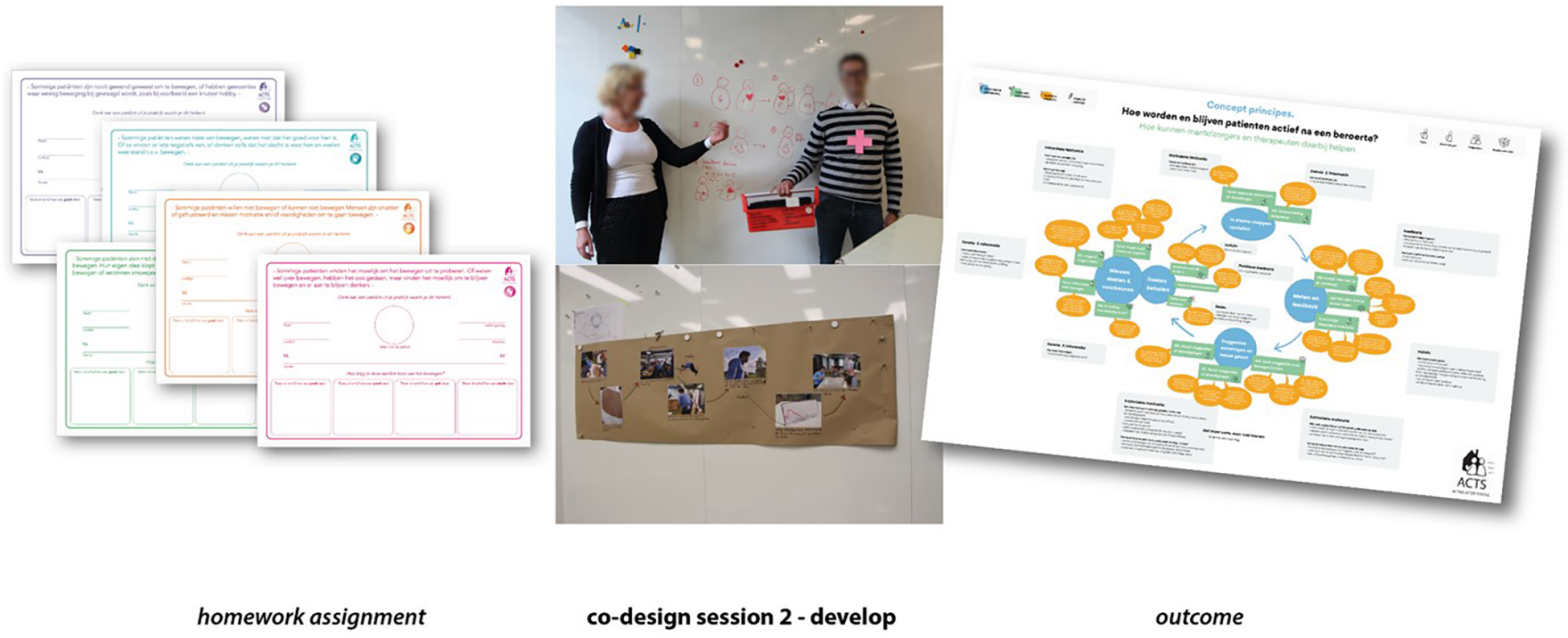
Co-design session 1—define.

Further insights were that therapists must have objective information on the walking activity of stroke survivors, inform stroke survivors about the benefits of being active, reassure them, and inform them of how much walking activity is needed to achieve their goals. Stroke survivors need to be given feedback on their walking activity levels to provide insight into their actual movement behavior. Measurement of walking activity must provide insight into the progress of stroke survivors (for the stroke survivor, carer, and therapist). Ongoing feedback on walking activity helps stroke survivors remember to be active throughout the day and provides information on whether the stroke survivor is moving toward their goal (doing more than yesterday/last week). Compliments from the therapist and the carer are welcome to encourage activities. Caregivers should share information with therapists about the stroke survivor being/becoming more active. Overall, it was thought that a comprehensive assessment by the therapist of the stroke survivor's environment/day routine/valued goals is necessary to inform a personalized intervention. Please see Figure 2.

### 3.3 Co-design session 3: deliver

For the interaction with the stroke survivor, generative communication tools were developed to support getting insights into the stroke survivor's usual daily routine, home and outdoor environment, (valuable) goals, and social network. Stickers with icons can be used to map out a stroke survivor's specific problems. Stickers with photographs can be used to map subconscious desires.

With regard to the screen belonging to the telemetric system, it was decided that the screen should be placed in the stroke survivor's living room as a reminder of the goals for the day. On the screen, time and date were added to also give it a function (clock) in the living room. In addition, the background picture on the screen represented a valuable goal picked by the stroke survivor. The number of steps to be taken for the day to achieve this valuable goal was depicted by a bar diagram on the screen.

**Figure 3:**
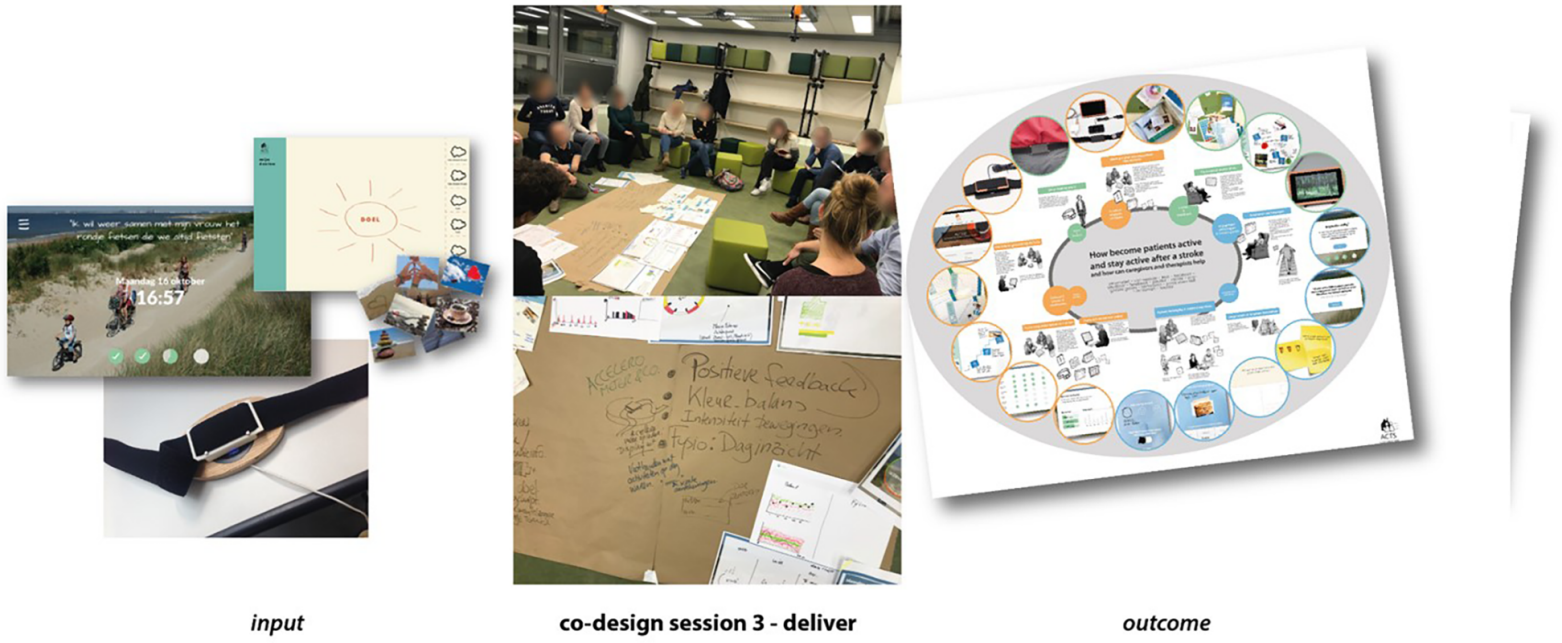
Co-design session 3—deliver.

A toolbox for therapists was developed that included the telemetric system (an accelerometer with a feedback screen on a small computer with a software system to transfer accelerometer data to the treating therapist's computer and to display walking activity to the stroke survivor in an attractive format) to be placed in the stroke survivor’s home and a series of communication tools to help get insights into the survivor's usual daily routine, the physical and home environment, the social network, and the survivor's goals (please see Figure 3 and Supplementary Appendix 3).

In terms of the proposed intervention, patients felt that the intervention would stimulate walking, and they liked getting feedback on how much had been done and how much remained to be done in terms of walking activity. They also felt that the intervention was not patronizing, but rather factual and that the steps outlined in it were positive and attainable. Therapists indicated that they liked the tools very much as they provided support in getting to know the patient better, which was felt to be especially important at the beginning of the intervention in order to understand why a patient was not walking enough. They felt that showing patients how much they had walked on a given day would contribute to a behavior change.

## 4 Discussion

This study used a co-design method to develop an intervention and tools that facilitate physical and exercise therapists in supporting an active lifestyle in stroke survivors, which is defined as a lifestyle that integrates daily walking activity with the day-to-day routine. To date, interventions for improving walking performance in stroke survivors have mostly focused on increasing walking capacity through training. Little attention has been paid to achieving a durable improvement in walking performance, with the aim of maintaining or improving gains made through physical interventions. In this context, the aims of this paper are (1) to describe the insights generated during the co-design process and (2) to describe the tools that were developed during the co-design process.

Several insights were gained during this process. The most important was the wide range of barriers and facilitators to walking performance for each stroke survivor, highlighting the need to provide personalized coaching. In order to be able to provide personalized coaching for individual stroke survivors, we found the following positive factors to be of importance: (1) A need for communication tools to explore the stroke survivor's context and goals; (2) Linking walking performance to meaningful (valued) goal setting, breaking down valued goals into smaller goals; (3) A need for an objective measurement of the stroke survivor's walking performance (accelerometer); (4) A need for objective feedback on the stroke survivor's walking performance (monitor); (5) A need for objective feedback to the therapist, to enable them to provide (positive) feedback on the stroke survivor's walking performance; (6) A need to stimulate interaction between the stroke survivor, carer, and therapist.

For objective measurement and feedback, we developed a system in which both stroke survivors and their treating therapists received objective information on the stroke survivors’ walking performance. We based this system on our previous work on the use of accelerometers in stroke survivors (36). We attempted to visualize stroke survivors’ goals in order to display them on the monitor as a reminder that walking performance is a means to a greater personal and valuable goal (prompts and cues).

We included BCTs that have been found promising in the literature (29), including goal-setting behavior, problem-solving, action planning, feedback on behavior, unspecified social support, and a credible source to improve walking behavior. In addition, we added self-monitoring of behavior and feedback on behavior by using accelerometers and framing–reframing by using the monitor that gave feedback on the number of steps walked. Using the latter behavior change techniques, we hoped that by giving stroke survivors objective feedback on their walking behavior, they would get an insight into their walking behavior and also a more realistic sense of the extent of their walking performance.

A strength of the study is that throughout the co-design process, stroke survivors, their carers, and therapists were involved, which allowed us to look at the problem both widely and deeply. This resulted in an enhanced understanding of the context of living at home, the role of the carer, and the community environment, as well as the importance of setting meaningful goals. We included evidence-based behavior change techniques and developed a system that allowed for an objective measurement of, and feedback on walking performance. This allowed therapists to give feedback on walking performance and for stroke survivors and carers to get direct feedback and information about the achievement of their daily goals on a monitor that also visualized stroke survivors’ goals. There were also some significant challenges. No one intervention would suit all stroke survivors. Therefore, we decided to design a toolbox, which we felt had enough elements for therapists to pick from to customize an intervention for a specific stroke survivor. As there is no existing/commercially available accelerometry system that provides raw data to researchers, considers the slower walking speeds of stroke survivors, and provides real-time feedback to stroke survivors and their therapists, we had to build such a system on our own. This took significantly more time and budget than we anticipated. In the end, we were not able to customize the system to the needs of the individual stroke survivors. There was a great deal of discussion among us on how to provide feedback visually to stroke survivors. In the end, we settled on a bar graph that filled up as stroke survivors came closer to their goal of the day. This system allowed therapists to “see” how much a stroke survivor had done in a day and set goals accordingly, but here too, the needs of therapists differed and we were unable to customize for all. Although we feel that this study illustrates how co-design facilitates the integration of contextual information into the intervention design to arrive at a prototype, the next step is to test the feasibility of this intervention in clinical practice.

## Data Availability

The raw data supporting the conclusions of this article will be made available by the authors without undue reservation.
