# Supplementary material for: Co-design of a walking activity intervention for stroke survivors
Source: Front Rehabil Sci. 2024 Jun 4;5:1369559. doi: 10.3389/fresc.2024.1369559 (PMC11183812; doi:10.3389/fresc.2024.1369559)
Supplement: Supplementary file 1 [file Table1.docx]

APPENDIX 1

**Behavioral lenses (a design tool as a way of looking at the world)**

### This method focuses on understanding the main problem to be addressed in an individual stroke survivor

The behavioral lenses are:

1. Habits and impulses: those things we do automatically without thinking (like sitting too much or exercising only when the sun shines).

2. Knowing and finding: the knowledge a stroke survivor has on the benefits of physical activity and the dangers of sedentary time or the personal conviction that exercise is good for other (younger) people,

3. Seeing and realizing: does the stroke survivor have appropriate insight into his/her movement behavior? Which excuses does the stroke survivor use to not be active?

4. To want and to be able: is the stroke survivor capable and motivated to be more active? Which activity would be suitable and appropriate to the stroke survivor’s preferences? Which are the barriers to being more active?

5. To do and keep doing: how do we keep the stroke survivor active? Is the “new” behavior more rewarding than the old behavior? Are there barriers to maintaining the desired behavior, is there social support?
