# Supplementary material for: Co-design of a walking activity intervention for stroke survivors
Source: Front Rehabil Sci. 2024 Jun 4;5:1369559. doi: 10.3389/fresc.2024.1369559 (PMC11183812; doi:10.3389/fresc.2024.1369559)
Supplement: Supplementary file 2 [file Table2.docx]

APPENDIX 2. Behavioral change techniques (BCT taxonomy v1) (37).

| **Technique** | **Description** | **Mechanism of action** |
| --- | --- | --- |
| Graded activity | Set achievable goals, increase in small steps | Skill: An ability or proficiency acquired through practice  Beliefs about capabilities; Beliefs about one’s ability to successfully carry out a behaviour |
| Problem solving | Analyze barriers, facilitators, and factors that influence behavior | Beliefs about capabilities |
| Credible source | Present verbal or visual communication from a credible source for or against the behaviour | Attitude towards the behaviour: The general evaluations of the behaviour on a scale ranging from negative to positive  General attitudes/beliefs: Evaluations of an object, person, group, issue or concept on a scale ranging from negative to positive |
| Social support (unspecified) | Advise on, arrange or provide social support (e.g., from friends, relatives, colleagues, buddies or staff) or non-contingent praise or reward for performance of the behaviour. It includes encouragement and counselling, but only when it is directed at the behaviour | Social influences: Those interpersonal processes that can cause oneself to change one’s thoughts, feelings or behaviours |
| Social support (practical) | Advise on, arrange, or provide practical help (e.g., from friends, relatives, colleagues, buddies or staff) for performance of the behaviour | Environmental context & resources: Aspects of a person’s situation or environment that discourage or encourage the behaviour  Social influences: Those interpersonal processes that can cause oneself to change one’s thoughts, feelings or behaviours |
| Goal setting (behaviour) | Set or agree on a goal defined in terms of a positive outcome of wanted behaviour | Intention: Conscious decision to perform a behaviour or a resolve to act in a certain way  Goals: Mental representations of outcomes or end states that an individual wants to achieve |
| Making an action plan | Prompt detailed planning of performance of the behaviour (must include at least one of context, frequency, duration and intensity). Context may be environmental (physical or social) or internal (physical, emotional or cognitive) (includes ‘Implementation Intentions’) | Behavioural cueing: Processes by which behaviour is triggered from either the external environment, the performance of another behaviour, or from ideas appearing in consciousness |
| Framing / reframing | Suggest the deliberate adoption of a perspective or new perspective on behaviour (e.g., its purpose) in order to change cognitions or emotions about performing the behaviour (includes ‘Cognitive structuring’) | Attitude towards the behaviour: The general evaluations of the behaviour on a scale ranging from negative to positive. |
| Prompts and Cues | Introduce or define environmental or social stimulus with the purpose of prompting or cueing the behaviour. The prompt or cue would normally occur at the time or place of performance | Memory, attention & decision processes: Ability to retain information, focus on aspects of the environment and choose between two or more alternatives  Environmental context & resources: Aspects of a person’s situation or environment that discourage or encourage the behaviour |
| Self monitoring behaviour | Establish a method for the person to monitor and record their behaviour(s) as part of a behaviour change strategy | Behavioural regulation: Behavioural, cognitive and/or emotional skills for managing or changing behaviour |
| Feedback on behaviour | Monitor and provide informative or evaluative feedback on performance of the behaviour (e.g., form, frequency, intensity, duration) | Motivation: Processes relating to the impetus that gives purpose or direction to behaviour and operates at a conscious or unconscious level |
| Review behavioural goals (outcome) | Review behaviour goal(s) jointly with the person and consider modifying goal(s) or behaviour change strategy in light of achievement. This may lead to re-setting the same goal, a small change in that goal or setting a new goal instead of (or in addition to) the first, or no change | Mental representations of outcomes or end states that an individual wants to achieve |
