# Supplementary material for: Co-design of a walking activity intervention for stroke survivors
Source: Front Rehabil Sci. 2024 Jun 4;5:1369559. doi: 10.3389/fresc.2024.1369559 (PMC11183812; doi:10.3389/fresc.2024.1369559)
Supplement: Supplementary file 3 [file Table3.docx]

APPENDIX 3: Interventions, based on the needs to provide personalized coaching by individualized intervention approaches and behavioral change techniques.

|  |  |  |  |
| --- | --- | --- | --- |
| **Intervention** | **Description** | **Insights** | **Techniques** |
| Generative communication tools  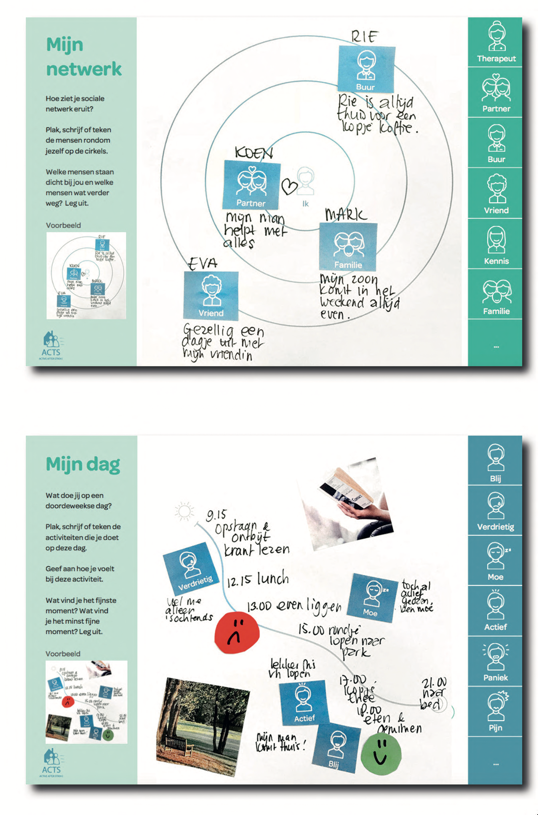 | Getting to know the stroke survivor, getting insight into a stroke survivor’s usual day, home and outdoor environment, (valuable) goals and the stroke survivor’s network.  Stickers with icons can be used to map out a stroke survivor’s specific problems. Stickers with photo’s can be used to map subconscious desires.  Insight in physical impairments, environmental limiting factors, lack of knowledge of the benefits of being active, (lack of) motivation and (old) habits can help to make plans to stimulate activity. | **Linking walking behavior to meaningful (valued) goal setting, breaking down valued goals into smaller goals.** | *Problem solving*  *Making an action plan* |
| Stroke survivor, carer and therapist interaction tools  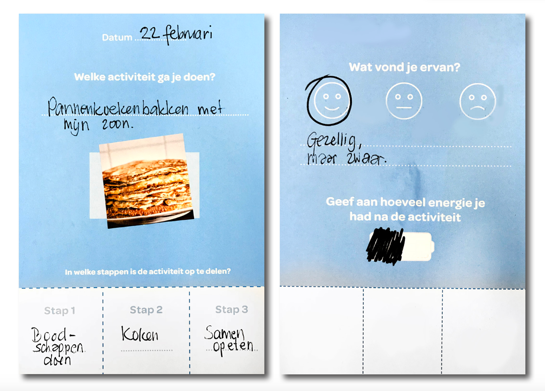 | Interaction between people helps a positive outcome of the intervention.  Therapist can inspire to move at home by getting the physical and social environment involved. Social support can make activity fun and feasible. Caregivers can also share concerns and ideas with the therapists and stroke survivor*.* | **A need to stimulate interaction between de stroke survivor, carer and therapist.** | *Credible source*  *Goal setting (behavior)* |
| Accelerometer screen  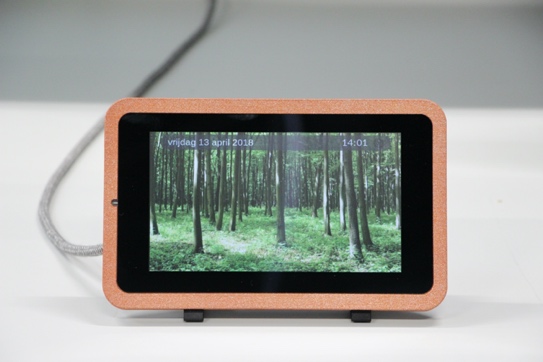 | Screen to be placed in living room, as a reminder and motivator of the goals for the day and taken steps to achieve this goal (for both stroke survivor and carer).  Background picture represents a valuable goal, reminder of the walking behavior being part of a higher goal.  Number of steps to be taken are shown by a bar diagram. | **A need for objective feedback on the stroke survivor’s walking behavior (monitor).**  **Linking walking behavior to meaningful (valued) goal setting, breaking down valued goals into smaller goals.** | *Feedback on behavior*  *Prompts and cues*  *Framing and reframing* |
| Accelerometry system  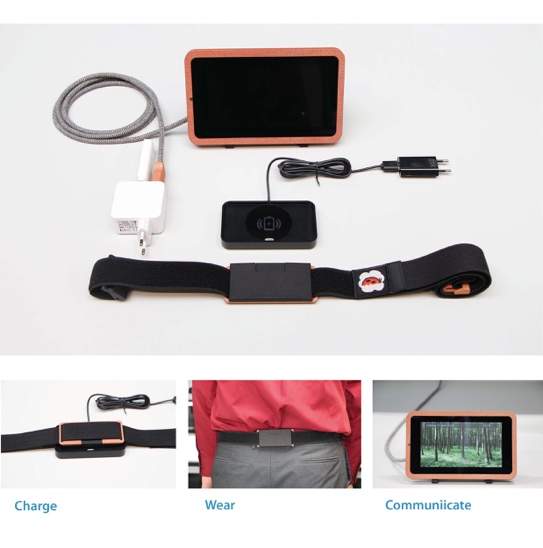 |  | **A need for objective measurement of the stroke survivor’s walking behavior (accelerometer).** | *Self monitoring of behavior* |
| ACTS website for physical therapists  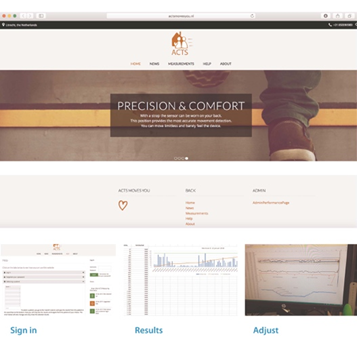 | A system to transfer accelerometer data to the treating therapist’s computer.  Therapists can use progress, compliments and suggestions to motivate stroke survivors*.* | **A need for objective feedback to the therapist, in order for the therapists to provide (positive) feedback on the stroke survivor’s walking behavior.** | *Feedback on behavior*  *Review behavioral goals* |
